# Developmental Changes of Human Neural Progenitor Cells Grafted into the Ventricular System and Prefrontal Cortex of Mouse Brain in Utero

**DOI:** 10.3390/cells12071067

**Published:** 2023-03-31

**Authors:** Maria Llach Pou, Camille Thiberge, Michiel Van der Zwan, Annousha Devi Govindan, Stéphanie Pons, Uwe Maskos, Isabelle Cloëz-Tayarani

**Affiliations:** 1 Institut Pasteur, Université Paris Cité, Unité de Neurobiologie Intégrative des Systèmes Cholinergiques, CNRS UMR 3571 “Gènes, Synapses et Cognition”, 25 Rue du Docteur Roux, 75015 Paris, France; 2 Collège Doctoral, Sorbonne Université, 75005 Paris, France

**Keywords:** xenografting, in utero transplant, human stem cells, human-induced pluripotent stem cells, chimeric mouse models, brain development, brain disorders

## Abstract

The transplantation of neural progenitors into a host brain represents a useful tool to evaluate the involvement of cell-autonomous processes and host local cues in the regulation of neuronal differentiation during the development of the mammalian brain. Human brain development starts at the embryonic stages, in utero, with unique properties at its neotenic stages. We analyzed the engraftment and differentiation of human neuronal progenitor cells (hNPCs) transplanted in utero into the mouse brain. The influence of the environment was studied by transplanting human NPCs within the lateral ventricles (LV), compared with the prefrontal cortex (PFC) of immunocompetent mice. We developed a semi-automated method to accurately quantify the number of cell bodies and the distribution of neuronal projections among the different mouse brain structures, at 1 and 3 months post-transplantation (MPT). Our data show that human NPCs can differentiate between immature “juvenile” neurons and more mature pyramidal cells in a reproducible manner. Depending on the injection site, LV vs. PFC, specific fetal local environments could modify the synaptogenesis processes while maintaining human neoteny. The use of immunocompetent mice as host species allows us to investigate further neuropathological conditions making use of all of the engineered mouse models already available.

## 1. Introduction

Experimental models of the human brain are needed for a better understanding of its development and the cellular and molecular dysfunctions that can occur during different stages of neuronal development, leading to autistic disorder, Asperger’s disorder, childhood disintegrative disorder, Rett’s disorder, and pervasive developmental disorder, and also other brain disorders such as schizophrenia, dementia, and Parkinson’s disease. There has been a significant progress in the field of human stem cells in recent years, with the main objective of reproducing the landmarks of neuronal disorders in vivo and decipher the altered underlying mechanisms in a context that resembles pathophysiological conditions. The development of stem cell transplantation models using human-induced pluripotent stem cells (hiPSCs) is also expected to offer new advances for regenerative medicine purposes, with the possibility of using specifically the allogeneic sources of these cells to produce autografts without the need for immunosuppression [1,2]. However, such clinical translations are still premature and more difficult to explore and adapt for therapeutic purposes. A large number of pre-clinical studies have already demonstrated the capacity for neuronal transplants to integrate within rodent brains and respond to guiding molecules and signals, under in vivo conditions [3,4]. In addition, it has been shown that transplanted pyramidal neurons derived from human embryonic stem cells can be specifically directed toward their appropriate sites of differentiation and maturation [5,6]. So far, the neuronal xenotransplants which have been obtained from hiPSCs include human neurospheres [7], human neuronal progenitor cells (hNPCs) [8,9], purified hiPSC-derived neurons with defined phenotypes [5,10], and, more recently, human brain organoids [11,12,13]. In parallel, experimental conditions have been set up which allow the integration of xenotransplants as single neurons within the mouse cortex where they can develop morphologically and functionally with a prolonged maturation period similar to that of the human developing brain [14]. Under such conditions, the transplanted neurons reach a more advanced stage of dendritic spine maturation as compared to those reported previously [15,16]. Therefore, the host brain seems to provide a permissive environment and the adapted cues for spinogenesis and synaptogenesis. In light of earlier reports, the transplantation paradigm used in conditions which allow the transplanted neurons to integrate as single cells represents a key determining factor during the final stages of the maturation and connectivity of these neurons within the host brain.

A major obstacle facing the transplantation of hiPSC-derived cells is the observed rejection of the grafts by the immune system of the host. Consequently, most transplantation models consist of grafting human cells into the brain of immunodeficient animals. The short-term immunosuppressive method allows for immunological tolerance with a significant improvement in the integration of transplanted iPSCs [17]. However, to increase the survival of transplanted cells, current methods are predominantly based on the use of transgenic immunodeficient rodent models. The brain is thought to possess an “immunological privilege” with a relatively large degree of permissible mismatching between the donor and the host [18,19,20]. In general, the main immune processes that occur during graft rejection involve the natural immunity of the host, which corresponds to the immune responses that naturally exist prior to transplantation. Some of these responses include the production of antibodies by B1 and B lymphocytes, mainly during the first weeks of life [21,22,23]. However, only the natural antibody of the IgG class can cross the barrier of placenta to assure fetal immunity [22,24]. Since innate responses are necessary to trigger the adaptative immune responses, some of the host immune responses cannot be fully active at the late embryonic stage or even at the early neonatal stages [24].

In the present study, we described a human neuronal-chimeric mouse by transplanting hNPCs into the brain of immunocompetent mice at the embryonic stage in utero. Our aim was to develop a new approach which prevents graft rejection and avoids bias due to the usage of immunodeficient mouse models. We specifically grafted NPCs within the mouse lateral ventricles at an embryonic stage at which only a small number of lymphocytes can infiltrate during brain development [25]. We used our previously characterized and established hiPSC-derived NPCs [9,10,11,12,13,14,15,16,17,18,19,20,21,22,23,24,25,26] to study some of the key morphological parameters at cellular levels at early stages of neuronal development. We showed that grafted neuronal precursors in the ventricles move towards their target regions during the first months following transplantation. When grafted into the embryonic neocortex at the same stage, we observed that these precursors integrated directly in the cortical areas with a more advanced maturation profile. 

## 2. Materials and Methods

### 2.1. Study Design and Ethics Statement

All methods were carried out in accordance with relevant guidelines and regulations. This protocol was approved by the Institut Pasteur Ethics Committee and the French “Ministère de l’Education Nationale, de la Recherche et de l’Innovation” under reference APAFiS #25221. Pregnant Swiss OF1 mice (Charles River Laboratories) were habituated at the animal facility for at least 3 days and surgeries were performed at the embryonic stage of E17.5.

### 2.2. Production of hiPSC and Their Derivation into hNPCs

The production of hiPSCs, their characterization, commitment to the neural lineage, and derivation of stable NPCs were performed according to Boissart et al. [27]. For this study, we used a control cell line from Coriell Biorepository (Coriell Institute for Medical Research, Camden, NJ, USA), the GM04603 (https://www.coriell.org/Search?q=GM04603, accessed on 25 January 2023) provided by Dr Alexandra Benchoua (I-Stem Institute, Evry) together with the derived NPCs [27]. For the initial experiments, we used the WTSli002-A line (https://cells.ebisc.org/WTSIi002-A/, accessed on 25 January 2023, Wellcome Trust, UK), as described in a previous study [28]. For the data presented here, we selected those obtained with the GM04603 iPSC line which has been fully characterized in vitro and in vivo in our previous studies [9,26,27]. 

The T25 flasks were coated under the flow hood with poly-ornithine (15 µg/mL in sterile H_2_O). After an overnight exposure at 37 °C, the flasks were washed three times with H_2_O. Then, the flasks were coated with laminin (1 mg/mL), diluted to 1/1000 in Advanced DMEM F12 medium, and incubated for at least 4 h at 37 °C. The NPCs were then plated at the density of 50,000 cells/cm^2^ in 5 mL of culture medium. The composition of the culture medium is described in Table S1 and prepared as previously described [9]. After the removal of the culture medium, fresh N2B27 medium containing 5 μL/mL of fresh laminin solution was added to keep the neurons attached and to avoid clumping. The medium was changed every 3 days. For the in vivo transplantation into the mouse embryos, we used hNPCs derived from the GM04603. 

### 2.3. Culture and Amplification of NPCs before Transplantation

**Figure 1:**
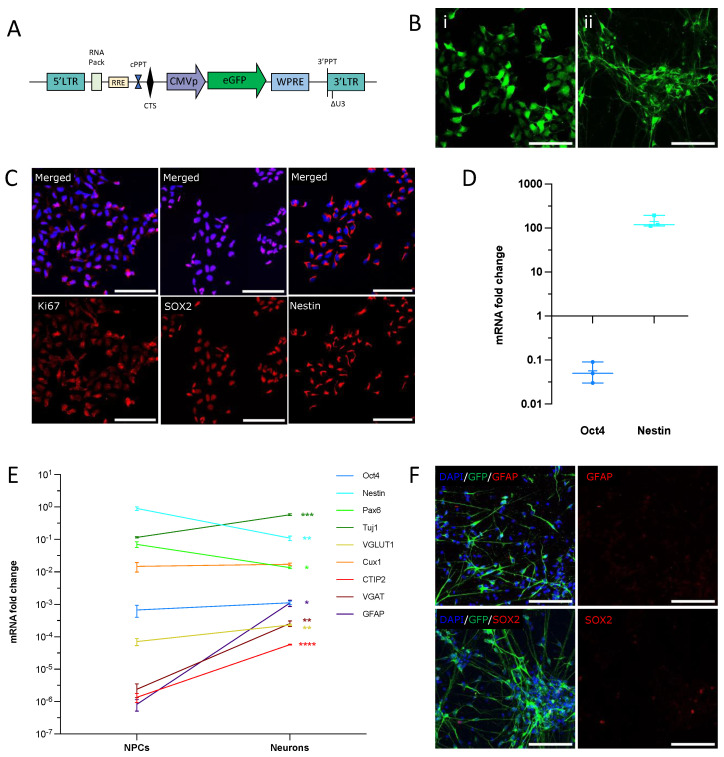
NPCs differentiate into immature cortical neurons in vitro. (**A**) Lentiviral construct expressing GFP used for NPCs labeling, adapted from [9]. (**B**) GFP is expressed in NPCs (left panel) and 30-day-old neurons (right panel). Scale bar = 100 µm. (**C**,**D**) NPCs express the neuronal progenitor markers Ki67, SOX2, and Nestin at passage 28 (**C**). The plot (**D**) shows mRNA expression fold-change as compared to human fetal brain. qPCR results were normalized to GAPDH and calculated with the ΔCT method (data are from three independent cultures). (**E**) mRNA relative expression of differentiation markers at NPCs and neuronal stage. Data are expressed as fold change to GAPDH calculated with the ΔCT method (* *p* < 0.05; ** *p* < 0.002; *** *p* < 0.0002; **** *p* < 0.0001, unpaired *t*-test). (**F**) Neurons do not express the astrocyte marker GFAP. Only a small proportion of cells remained SOX2 positive after 30 days of differentiation. Scale bars = 100 µm.

The culture and amplification of NPCs were performed in sterile conditions. For their labeling, NPCs were transduced with a CMV-GFP-lentiviral vector to obtain green fluorescence, as described previously [9,29]. Lentiviral vectors were prepared according to a published protocol [30]. The map of the CMV vector is detailed in Figure 1A. Briefly, NPCs were thawed in T75 flasks at a density of 50,000 cells/cm^2^ in 25 mL of NPCs medium supplemented with Rock Inhibitor Y27632 (10 μM). The NPCs were transduced with 99.5 ng of lentivirus (p24) in a 6-well culture plate filled with 500 μL of fresh culture medium for 3 h. The volume was completed to 2 mL, incubated for the next 48 h, and then rinsed with the culture medium. All media and products are described in Thiberge, Llach Pou et al. [31] and are summarized in Table S1. 

### 2.4. In Utero Transplantation of Mouse Embryos

**Figure 2:**
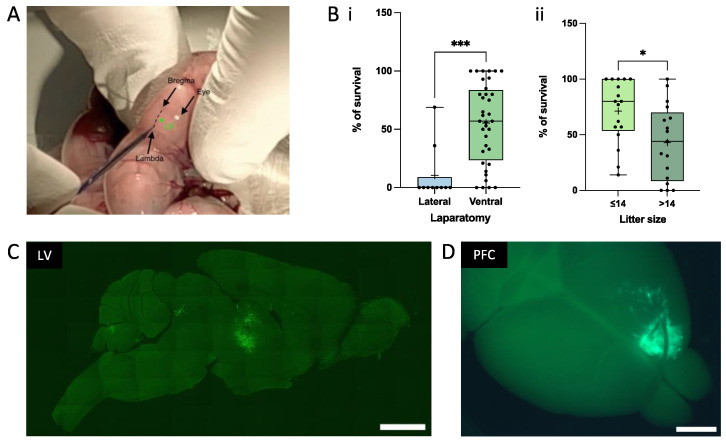
The in utero technique. (**A**) Illustration of the site of injection for Lateral Ventricle (LV) surgery. (**B**) Pup survival as a function of the laparotomy technique (Bi) and litter size (Bii). Pup survival is higher when the ventral laparotomy is performed on mice that have less than 14 embryos. Each point of the boxplot corresponds to the survival percentage in one litter. (* = *p* < 0.05; *** = *p* < 0.001, unpaired *t*-test). (**C**) Sagittal picture of a brain section at 1 MPT into LV taken with an epifluorescence microscope. Cells are labeled with GFP. (**D**) Illustration of a graft within the PFC at 1 MPT taken with an epifluorescence macroscope. Scale bars = 2 mm.

Before their transplantation, the cells were kept in a water bath at 37 °C. The injection solution also contained 20 mM EGTA to avoid cell aggregation upon transplantation [14,31,32]. The transplantation protocol has been previously detailed [31]. Briefly, 2 μL containing 2 × 10^5^ cells were injected within 1 lateral ventricle or within 1 side of the PFC of E17.5 embryos. The injection within LV is depicted in Figure 2A and the injection within PFC was described previously using mouse neonates (P0-P1) [8,9]. Thirty minutes before surgery, buprenorphine (0.05 to 0.1 mg/kg) was injected subcutaneously at the back of the neck of the pregnant mouse for initial analgesia. The pregnant mouse was then anesthetized with isoflurane and oxygen and placed on a heating pad connected to the anesthesia unit. A drop of ocryl gel was applied on each eye. The abdomen was shaved locally for ventral laparotomy and lidocaine gel was applied locally before and after the laparotomy. A sterile towel drape was positioned on the mouse abdomen. An incision of 1 to 1.5 cm was made in the lower abdomen in a manner to cut both the skin and the thin fascia layer. The whole uterus was carefully extracted from the abdomen. During surgery, drops of sterile PBS kept at 37 °C were consistently applied on the exposed fetuses. The sharp glass pipette was carefully inserted through the fetal skull, and cells were injected into each fetus, except the first one to be delivered on each uterine horn (Video S1). After the injection, the pipette was carefully withdrawn. Then, the uterus was reentered into the abdominal cavity. The peritoneal cavity was rehydrated with 0.5 to 1 mL of sterile PBS to replace any fluid loss during the surgery. Then, the incision was closed with two types of sutures: for fascia and skin, we used sterile resorbable sutures (ProleneTM, EH7471, Ethicon, LLC, Issy les Moulineaux, France) and non-resorbable sutures (Coated Vicryl, V302, Ethicon, LLC), respectively. After the surgery, the pregnant mouse was positioned on a warming pad until the mouse was fully awake. Analgesia treatment (acetaminophen 4 mg/20 mL) was administered in the drinking water for 3 to 5 days after the surgery. Hydrogel was also given to the mice for one day after the surgery. We could observe that the presence of EGTA significantly reduced the cell aggregation and increased the possibility of identifying single cells within the mouse brain, as described previously [14]. 

### 2.5. Quantitative Real-Time PCR (qRT-PCR) 

The total mRNA from NPCs and neuronal cultures was extracted with the RNeasy Plus Mini Kit (ref. 74034, Qiagen). cDNA was generated using the High Capacity cDNA Retro-Transcription Kit, according to the manufacturer’s instructions (Ref. 4368814, ThermoFisher). For mRNA expression level quantification, real-time PCR was performed on the Applied Biosystems 7500 Real-Time PCR System. UDG activation (1 cycle, 2 min, 50 °C) and polymerase activation (1 cycle, 10 min, 94 °C) were followed by 40 cycles of denaturation (15 s, 95 °C) and anneal/extend (1 min, 60 °C). The PCR reactions contained 500 nM of each primer, 0.5 × PowerUP SYBR Green Master Mix (2×) (A25742, Applied Biosystems), and 20 ng of cDNA in a final volume of 10 μL. The primers were manually designed to amplify mRNA only (at least one of the primer pairs spans the exon-to-exon junction). The list of primers is presented in Table S2. The specificity of the primers was verified with a melting curve and agarose gel electrophoresis of the PCR confirmed amplicon size. The mRNA expression level was expressed as the fold change to the housekeeping gene GAPDH (Figure 1E) using the 2^−ΔCT^ method or normalized to GAPDH and compared to a control condition using the 2^−ΔΔCT^ method (Figure 1D) [33].

### 2.6. Immunofluorescence (IF)

Immunofluorescence analysis on brain sections was performed at 1 and 3 MPT, according to our previously described protocol with slight modifications [9,31]. Animals received a lethal dose of a mixture of Ketamine (Imalgen^®^, 100 mg/kg, Merial) and Xylazine (Rompun^®^, 10 mg/kg, Bayer), and were transcardially perfused with 15 mL of DPBS, followed by 40 mL of ice-cold paraformaldehyde (PFA) (4%) in 0.1 M phosphate buffer (pH 7.4). The brains were dissected and post-fixed overnight at 4 °C in 4% PFA. In total, 40 μm coronal sections were obtained using a microtome (Leica SM2010 R), collected in 12-well culture plates, and stored in DPBS + Azide (0.02%) at 4 °C until use. The free-floating sections were rinsed twice in PBS (pH 7.4) and then incubated in a DPBS solution containing 0.2% Triton X-100 and 10% goat or horse serum at room temperature (RT) for 30 min to block nonspecific binding. An extra step of blocking using MOM (Ready Probes ^TM^ Mouse on Mouse IgG Blocking Solution (30×), R37621, Invitrogen) was added if a mouse primary antibody was used. The primary antibodies, diluted in blocking solution, were added overnight at 4 °C. The list of all antibodies and their dilutions is presented in Table S3. An additional incubation with DAPI diluted in PBS was performed for 10 min at room temperature [29].

### 2.7. Mapping and Semi-Quantification of Cell Bodies and Projections 

Fluorescence images of whole brain slices were captured using a laser-scanning confocal microscope (Axio Scan.Z1, Inverted LSM 700 from the Histology Platform at Institut Pasteur). Three different tile images at 20× magnification from three independent transplanted animals were taken per condition and analyzed by two distinct experimenters. To overcome the limits of conventional manual counting of labeled cells, we implemented a novel analysis pipeline allowing for the reconstruction of the expected shape of each brain slice (Figure S1). This method reduces any bias in brain mapping that may result from slight variations of slicing conditions along the anterior-posterior axis of the brain. This pipeline allows the precise mapping of single neuron positions and projections by automatically setting a grid of squares of ~500 µm^2^. The Allen Brain Atlas was used as a reference for the precise matching of experimental brain slices with the expected brain cartography. A grid of squares was applied to each section and the cells were manually counted. Semi-quantification of the cell bodies and projections was performed, and color codes were applied. To avoid bias due to possible graft heterogeneity, we selected brains with similar grafting for all quantifications.

### 2.8. Quantification of Dendritic Spines 

We reported a protocol for the imaging of dendritic spines after immunofluorescence in a video journal [29]. Briefly, confocal images were acquired with a confocal laser-scanning microscope (LSM 700, Zeiss) using a 40× oil NA = 1.3 objective, and a 488 nm laser for GFP excitation. A Z spacing of 200 nm was used for Z-stack acquisitions. A semi-automatic tracing of dendrites and automated segmentation of spines was performed using the Filament Tracer module of Imaris 9.5 software (Bitplane AG, Zürich). The spine categories were defined by their morphology as follows: Stubby: length < 1 μm; Mushroom: max-width-head > min-width-neck*2; Thin: length (spine) < 2 and mean-width-head ≥ mean-width-neck; Filopodia-like: length ≤ 4 μm and >1 μm, by the MATLAB, version 2022a. Spines were quantified at a 20 μm distance from the soma [34]. The 3D images and videos (.mp4 files) of spines were obtained from Z-stacks of confocal images and processed using the animation functions in Imaris software to zoom and navigate around the dendrites and spines. For LV transplantation, we did not include hNPC-derived neurons that were present in matter tracts or present in non-neuronal environments, but quantified only those present in cortical areas.

### 2.9. Statistical Analysis

Statistical analyses were performed using GraphPad Prism Version 9 software (GraphPad, San Diego, CA, USA) and are reported in each figure legend. Confidence levels of 95% were used.

## 3. Results and Discussion

### 3.1. hNPCs Differentiate into a Homogeneous Population of Immature Neurons In Vitro

For this study, we used a well-characterized human iPSC line for which derived hNPCs were GFP-labeled, as we described previously [9,26,29]. We did not exceed the number of cell passages that could have altered the NPCs’ amplification and proliferation [10]. We first checked that these cells could differentiate into a homogeneous population of cortical neurons in vitro, as we have shown previously. Our data indicate that human NPCs and hNPC-derived neurons express similar levels of green fluorescence (GFP), with a more mature shape after 30 days of in vitro differentiation (Figure 1A,B). The hNPCs displayed positive immunoreactivity for the cortical radial glial markers SOX2 and Nestin, and for the proliferation marker Ki67 (Figure 1C). The mRNA level of OCT4, a marker for undifferentiated cells, was decreased when compared to the fetal brain, while Nestin mRNA was increased (Figure 1D). Only a few cells expressed SOX2 in the cultures of 30-day-old neurons (Figure 1F), indicating successful neuronal differentiation. Figure 1E presents the comparative expression of differentiation (CTIP2, Oct 4, Pax 6, and Nestin), neuronal (VGLUT1, VGAT, Tuj1, Cux1), and astrocytic (GFAP) markers in NPCs and neuron stages. mRNA was measured by RT-qPCR and the levels were normalized to GADPH. The Nestin mRNA levels decreased in the 30-day-old neurons, as compared to the NPCs, while those for Oct 4 remained very low. The expression of CTIP2, the vesicular GABA transporter (VGAT), TUJ1, GFAP, and, to a lesser extent, Cux1 were increased in neurons as compared to NPCs (Figure 1E). CTIP2 is a transcription factor expressed in the cortical layers V-VI neurons, which has also been linked to axonal growth and the establishment of the cellular architecture of the striatum, in vivo [35,36]. VGAT, the vesicular GABA transporter, has been shown to play a role in the embryonic development [37]. Although our protocol provides a large majority of excitatory cells [27], we cannot exclude some VGAT expression during the earlier stages of neuronal differentiation. In parallel, we did observe higher levels of VGLUT1 mRNA as compared to VGAT, in NPCs and similar levels in neurons. A slight increase in VGLUT1 mRNA was also found in neurons as compared to NPCs. Tuj1 is a marker of neuronal lineage, expressed at early stages of maturation as well as in mature neurons. This is in accordance with a slight increase in its expression in neurons, as compared to NPCs. The GFAP mRNA levels were increased in neurons, but this increase was not correlated to the increase in protein levels since no GFAP could be detected at the same stage of neuronal maturation (Figure 1F). In accordance with Boissart et al. [27], Cux1 mRNA was higher than CTIP2 mRNA in both conditions. As expected for more mature neuronal cells, Pax6 and Nestin expressions were slightly decreased in the neurons as compared to the NPCs.

### 3.2. In Utero Grafting of Human iPSC-Derived NPC into Embryonic Mouse Brain 

The xenotransplantation of neural progenitors represents a very useful method to study the contribution of the intrinsic mechanisms and their dynamic crosstalk with the host factors that control and regulate cell migration and differentiation within the developing brain [38]. The regional determination of neural progenitors is an early event, which is still ongoing at the stage of E17.5 [39]. During the course of this study, we first aimed at demonstrating that human NPCs could integrate, differentiate, and migrate after their transplantation into the E17.5 mouse brain, in utero. For that purpose, we used experimental conditions which allow the human precursors to integrate as single cells, which will, in turn, result in a coordinated morphological and physiological development of their dendritic spines similarly to our previously reported observations in neonatal xenotransplantations [14]. In order to target the earlier stages of development, we set up a technique which allows for the in utero transplantation of NPCs into the LV and PFC embryonic regions. We first compared the percentage of pups’ survival between lateral and ventral laparotomy and observed that ventral laparotomy was the most adapted method, at least in the case of large litters, such as those expected with Swiss OF1 mice (Figure 2Bi). The percentage of pups’ survival was also significantly increased when the litter did not exceed 14 pups (Figure 2Bii). Figure 2C illustrates that a large number of cells can survive and integrate into the periventricular areas, as well as the whole ventricular system, including the third ventricle, when grafted into the LV, as shown at 1 MPT (left panel). Figure 2D also shows a direct view of an in utero transplant within the PFC, as shown at 1 MPT (right panel). The scanned cortical transplant can be observed directly within the transplanted hemisphere. This would allow the exclusion or rejection of any abnormal transplant before further analysis. Regardless of the mechanisms that may be involved locally, these initial observations indicate that progenitor cells are able to survive and integrate within distinct brain regions.

### 3.3. Achievement of a Stable Cell Engraftment Using Immunocompetent Mice and Maturation Pattern of Axonal Projections up to 3 MPT

**Figure 3:**
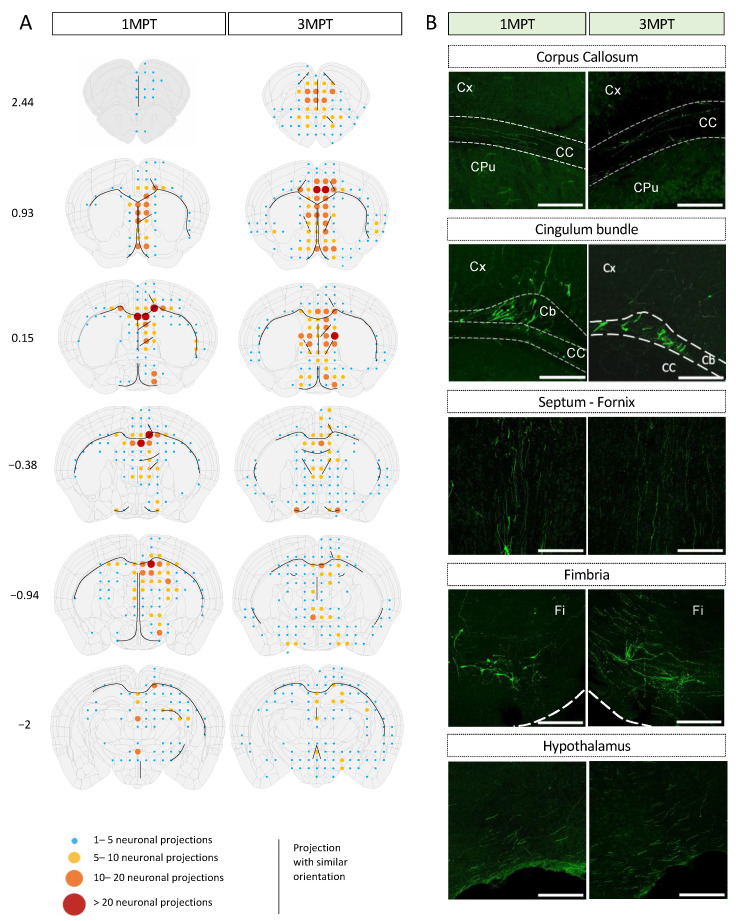
Mapping of neuronal projections from transplanted human cells within the LV of the embryonic mouse brain at 1 MPT and 3 MPT. (**A**) Camera lucida drawings are according to Allen Brain Atlas (https://mouse.brain-map.org/, accessed on 25 January 2023). Image credit: Allen Institute. The densities of neuronal projections are depicted using a code-color schematic representation. Results are calculated as the mean value from three independent mice. Two to three brain sections were analyzed for each coordinate (Bregma, indicated in mm) and per mouse. (**B**) Representative images of GFP+ projections at 1 MPT and 3 MPT in fiber tracks and brain regions such as the cortex (Cx), corpus callosum (CC), cingulum bundle (Cb), septum/fornix, fimbria (Fi), and hypothalamic region. The illustrated data are from the three representative brains. Scale bars = 200 µm.

**Figure 4:**
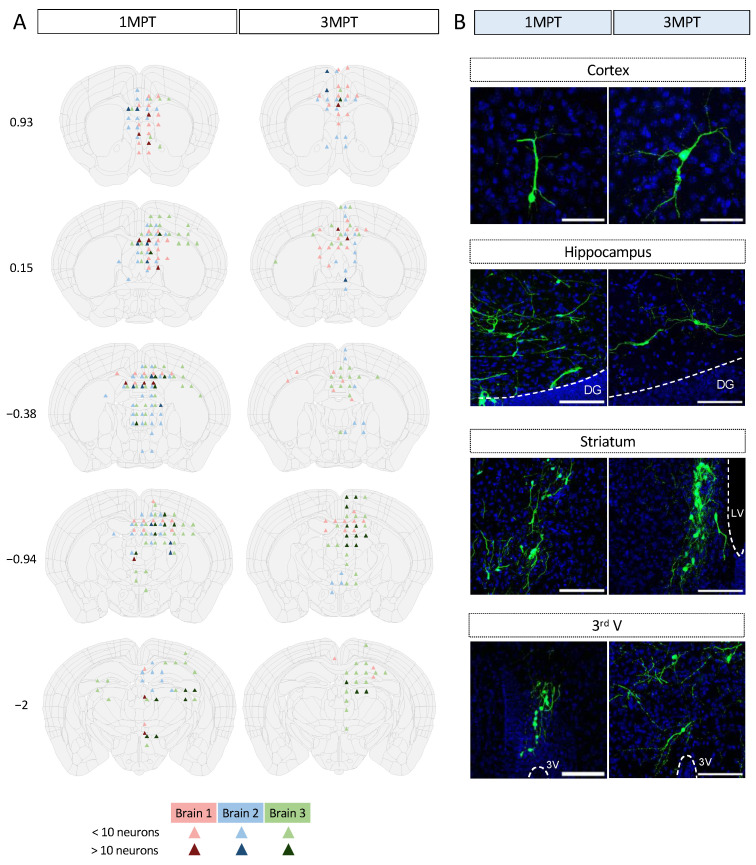
Mapping of neuronal somas from transplanted human cells within the LV of the embryonic mouse brain at 1 MPT and 3 MPT. (**A**) Camera lucida drawings are according to Allen Brain Atlas (https://mouse.brain-map.org/, accessed on 25 January 2023). Image credit: Allen Institute. Two brain sections were analyzed for each coordinate (Bregma, indicated in mm) and per mouse. Each brain analyzed is represented in a different color and the number of neuronal somas is represented in a color-code manner. For each brain and coordinate, 40-µm thick images were used and the counted values summed. (**B**) Representative images of brain regions where the grafted human cells tended to migrate.

According to our differentiation protocol, we expect hNPCs to differentiate mainly into glutamatergic pyramidal excitatory neurons, morphologically characterized in vivo by a conical “tear-shaped” soma, an apical dendrite, and basal spiny dendrites. Pyramidal neurons can extend their axon at long distances from the cell body. It has recently been shown that axons can also originate from dendrites in mammals and in humans to a lesser extent [39]. Under our experimental conditions, we could not detect any axon arising from dendrites. Detailed mapping of somas and axonal projections originating from ventricular transplants are depicted in Figure 3 and Figure 4. hNPCs were transplanted in utero within the mouse LVs. The distributions of cells and their projections were quantified independently by two (somas) and three (projections) observers, as described in the experimental procedures section. The integration of GFP-labeled cells, their survival, maturation, and phenotypic fate over time was followed up to 3 MPT. The transplants did not increase the brain surface, nor did they remain confined within the LV. We could easily identify GFP+ neurons within different mouse brain regions with a typical pyramidal morphology (Video S2; Figure 4). The semi-quantitative analysis at 1 MPT and 3 MPT indicates the presence of variable densities of GFP+ projections within distinct brain areas. Many GFP+ projections were detected within the brain structures close to the external walls of the lateral and third ventricles. As we were able to observe at different levels of the host brain, the GFP+ projections reached their highest densities in the corpus callosum, septum, caudate putamen, hippocampus, and thalamic nuclei. The projections were distributed both ipsi- and contralaterally to the same extent at 3 MPT (Figure 3). These findings are in accordance with those reported previously after in utero grafting of mouse neurons into the embryonic rat brain [40]. The two hemispheres are connected by commissural fiber systems, including the corpus callosum and hippocampal commissures, which were enriched in axonal projections (Figure 3). The two LVs are separated from each other by a thin vertical sheet [41] but they communicate with the third LV in which projections were also found (Figure 3). Perpendicular projections could also be detected between the ventricular wall dorsal to the CA1 hippocampal brain region of the host. GFP+ projections were also detected to a lesser extent in the cortex and the subcortical areas, including the hippocampus and thalamus (Figure 3). The pattern of projections was slightly different from that we previously observed under ventricular transplantation in neonates [9]. More precisely, we did not observe any visible axonal projections along the rostral migratory stream, nor the presence of cells within the olfactory structures. This indicates that under in utero grafting conditions, the NPCs endogenously predisposed to become excitatory cells did not significantly follow the same pathways promoting the migration of interneurons to olfactory structures. Espuny-Camacho et al. reported a widespread distribution of cell projections [5]. However, the distribution of GFP-labeled cell bodies has not been fully investigated in xenotransplantation studies with human iPSC-derived neurons. This may be due to the fact that a precise quantification of GFP-labeled cell bodies within a specific brain structure requires volumetric analysis among several transplanted brains. It should also be noted that small variations in the cell location and integration at the embryonic stage could lead to larger variations in the adult brain. It should be noted that assessing the number of grafting cells would require the use of the same grafted brain for analysis at different time points, which is not technically possible by standard methods. Such quantifications have not been reported so far. One of our objectives for this study was to determine the capacity of cells to survive upon their in utero transplantation, while keeping their migratory properties over time. Despite some variabilities among the three independent brains which were analyzed, we observed similar patterns of cell distributions in the regions close to the ventricular system at 1 MPT (Figure 4). At 3 MPT, the transplanted cells displayed the properties needed to migrate into more distant regions, which include lateral and medial septum, caudate putamen, retrosplenial, motor and anterior cingulate cortex, hippocampus, some thalamic nuclei, and preoptic areas (Figure 4). Similar patterns were observed among the 3 brain samples which were evaluated independently at 1 MPT and 3 MPT. During neuronal migration, NPCs are expected to adapt their migration and final location by following well-known migratory pathways [42]. Our results indicate that at least some of the intrinsic migratory properties of human cells remain unchanged while they develop in the murine host in a similar manner among independent transplanted mice. Our data also indicate that the number of GFP+ cells remained similar between 1 MPT and 3 MPT in 3 independent experiments and according to the quantification of 3 brain sections per region and brain (Figure 4). 

In contrast to previous studies, we did not observe any significant graft rejection at 1 MPT despite the use of an immunocompetent host, as previously observed by other authors [43]. We, therefore, believe that under our experimental conditions, human-transplanted cells might express some specific antigens at least prior to the maturation of the mouse immune system, thereby preventing the rejection of a transplant by the host immune system. Additionally, it has been reported that iPSC-derived NPCs display low immunogenicity [44]. More recently, immature neural precursors have also been reported to partially suppress the proliferation of murine CD4 and CD8 T cells involved in the adaptative immune responses [43]. Such adaptative immune responses are under the control of the host natural immunity which is not fully active at late embryonic or even at early neonatal stages [24]. Since the brain has been considered immunologically to be a privileged organ [44,45], these findings could provide further explanations and help understand the reasons for the absence of graft rejection in our experiments. 

### 3.4. Analysis of Human Cell Neuronal Phenotypes after in Utero Transplantation at 3 MPT

**Figure 5:**
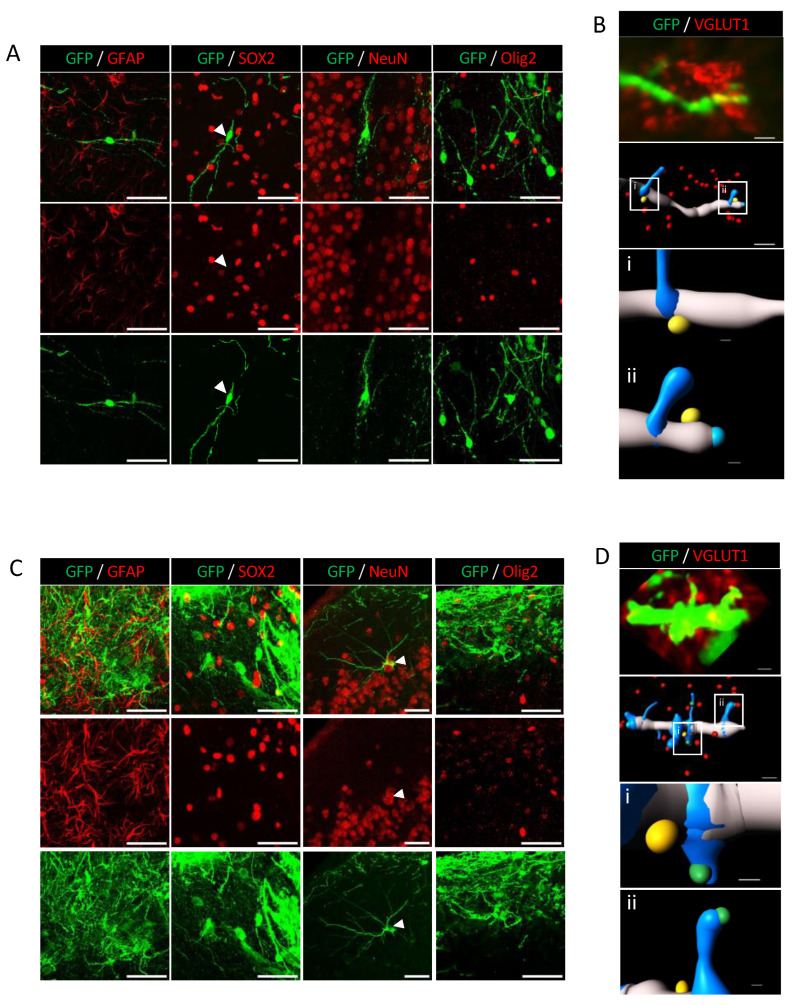
NPCs differentiate into glutamatergic neurons at 3 MPT. Immunofluorescence staining of NPC-derived neurons by GFAP, SOX2, NeuN, and VGLUT1 at 3 MPT after LV (**A**,**B**) and PFC (**C**,**D**) transplantation. Left to right panels, no positive human cells were found for GFAP, Sox2, NeuN, and Olig2 (no double-labeled cells). (**B**). Punctiform labeling of dendritic segment was observed for VGLUT1, clearly illustrated by a 3D zoomed-in image showing distinct VGLUT1 punctae represented in yellow (Bi, Bii). (**C**). Left to right panels, no positive human cells were found for GFAP, Sox2 and Olig2. Positive labeling for NeuN marker showing the nuclei of pyramidal neurons within the PFC (arrowhead). (**D**). Punctiform labeling of dendritic segment and associated spines was observed for VGLUT1, clearly illustrated by a 3D zoomed-in image showing distinct VGLUT1 punctae represented in yellow (dendrite) and green (spines) (Di, Dii). Scale bars = 50 µm (GFAP, SOX2, NeuN and Olig2 images), scale bars = 2 µm (reconstituted VGLUT1 images), and scale bars = 0.3 µm (zoomed images Bi, Bii, Di, Dii).

During brain development, specific neuronal subtypes are produced in a specific temporal manner. The protocol we used for the derivation of human NPCs into cortical pyramidal neurons was adapted from Boissart et al. [27]. It allows the NPCs to differentiate into large proportions of excitatory neurons (≅80%), mainly from the cortical layers II-IV, and, to a lesser extent, into GABAergic cells (≅20%) in vitro. Using the immunostaining approach, there was an absence of SOX2 and GFAP labeling of human cells after in utero transplantation at 3 MPT, and in LV and PFC conditions. We did not observe any SOX2 or GFAP expression in the differentiated cells in these conditions (Figure 5A–C, Video S3). Our data are consistent with those obtained in vitro (Figure 1) and indicate that human cells have lost their capacity to proliferate without differentiating into astrocytes at 3 MPT after LV and PFC transplantation. It has been previously shown that sequential mechanisms may exist and confer young neurons to differentiate into astrocytes [46]. Therefore, one cannot exclude some derivation of human cells into astrocytes at later stages of neuronal differentiation as we could observe in vitro at 40–45 days of differentiation using the same iPSC line together with two other iPSC lines [26]. We analyzed the expression of the neuronal nuclear marker NeuN, a selective marker of postmitotic neurons. We could not clearly identify isolated mature neurons that co-expressed NeuN and GFP at 3 MPT after LV transplantation and in all of the neuronal migration brain subregions (Figure 5A). By contrast, NeuN was clearly expressed in the human NPC-derived pyramidal cells after PFC transplantation, as illustrated in Figure 5C. As a marker for glutamatergic neurons, we selected the vesicular glutamate transporter VGLUT1, which is expressed in all of the neuronal migration brain regions [47]. We could detect the expression VGLUT1 in NPC-derived neurons after LV and PFC transplantation at 3 MPT. The 3D reconstruction and zoomed-in visualization of dendrite segments and spines allowed us to demonstrate at a microscale the colocalization of VGLUT1 with GFP labeling (Figure 5B–D). We could observe that VGLUT1 was only expressed at the levels of dendritic shafts after LV transplantation but also at the spine levels after PFC transplantation (Figure 5D; Videos S4 and S5). We also analyzed the expression of the oligodendrocyte marker Olig2 and did not detect any colocalization of Olig2 with GFP labeling (Figure 5A,C). For analysis of progressive cell death over time, we tested the expression of apoptosis-related caspase-3 protein at 1 MPT and 3 MPT (Figure S2). Our results did not show any significant caspase-3 labeling at the two time points. To evaluate the maturation of the transplanted cells between LV and PFC transplants, we analyzed the differential expression of the NeuN marker for mature neurons with that of β3-tubulin, a marker of the early phases of neuronal differentiation (Figure S3). Our results strongly suggest higher levels of neuronal maturation with increased glutamate levels neurotransmission in human NPC-derived neurons transplanted in PFC, as compared to those transplanted in LV. 

**Figure 6:**
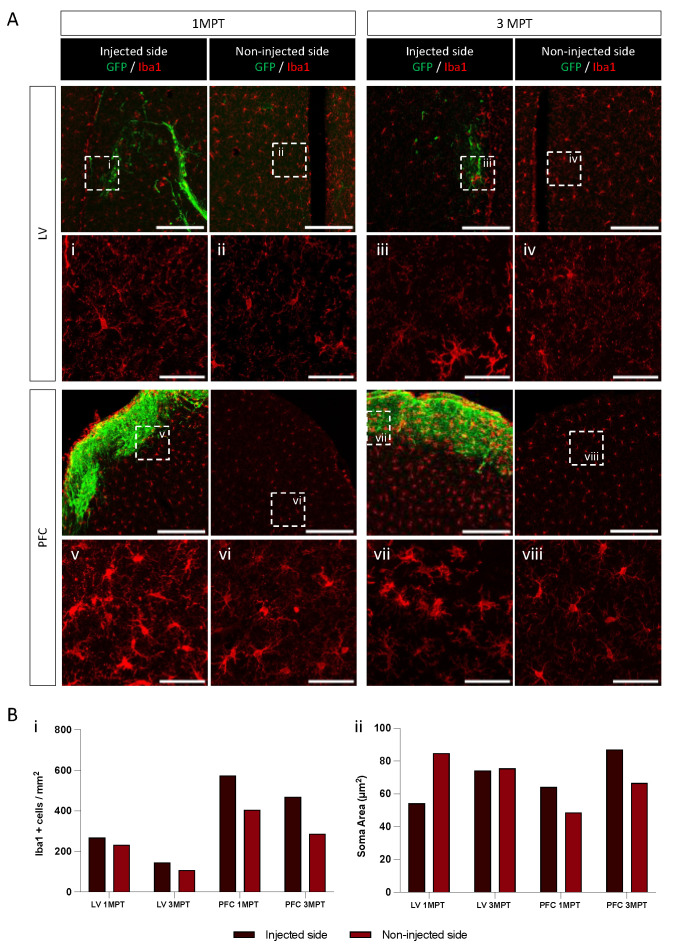
Microglia recruitment around the grafted area, related to Figure 5. (**A**) Confocal images at 1 MPT and 3 MPT of the injected and non-injected sides for LV- and PFC-transplanted mice. The transplanted cells are labelled with GFP, and microglia is shown in red. Pictures i–viii show the higher magnification of the area for the analysis of microglia morphology on one representative brain for each condition. Scale bar = 200 µm and 5 µm for i–viii. (**B**) Plot showing microglial density (Bi) and soma area (Bii) for each condition. Quantification was performed on the 20× images, using the MorphoLibJ plugin in Fiji.

Microglia have been described as a regulator of the proliferation of neural precursors cells in the developing cerebral cortex [48]. They play a role in synaptic pruning and synaptic density and can rapidly detect changes in the brain environment [49,50]. Using single-cell and spatial transcriptomic profiling, it has been recently shown that the neuronal identity can influence microglia densities [51]. During the course of this study, we analyzed the potential enrichment of mouse microglia at transplantation sites at 1 MPT and 3 MPT after LV and PFC transplantations. For this purpose, we used Iba1 labeling to identify the presence of microglia cells at injected and non-injected hemispheres (Figure 6). Interestingly, we could observe differences in microglia labeling under all of the conditions tested. The number of Iba1+ cells remained within the same order between the injected and non-injected sites in LV transplantation at both 1 MPT and 3 MPT. A slight decrease in cell numbers was observed in the non-injected site, where the transplanted cells are expected to be less present, at least at 1 MPT (Figure 6). As compared to LV, the PFC transplantation induced higher levels of Iba1+ cells, initially surrounding the transplanted area at 1 MPT, and then integrating within the transplanted area at 3 MPT. In the PFC region, the level of resident microglial cells was twice higher as high as that of the LV region. These observations are in agreement with previous reports on the regional heterogeneity of microglia within specific brain regions, among rodent brains [52]. We also analyzed the morphology of microglial cells as an index of their reactivity [53]. The small cell somas and large territories occupied correspond to a lower activity state, while cell body enlargement and more irregular cell shape correspond to a high activity [54]. After LV transplantation, we did not observe any significant variation between 1 MPT and 3 MPT, and between the ipsilateral and contralateral sites (Figure 6). After PFC transplantation, we observed a slight increase in the soma areas between 1 MPT and 3 MPT, and between the injected and non-injected sites. Microglia are expected to regulate the survival of neuronal cells without excluding a possible phagocytosis of dead cells and debris that may result during PFC transplantation, where the high densities of human cells may influence their glial environment. Further studies are needed to examine and confirm this hypothesis.

### 3.5. Comparative Analysis of Dendritic Spines in LV- or PFC-Transplanted Human Cortical Pyramidal Neurons 

**Figure 7:**
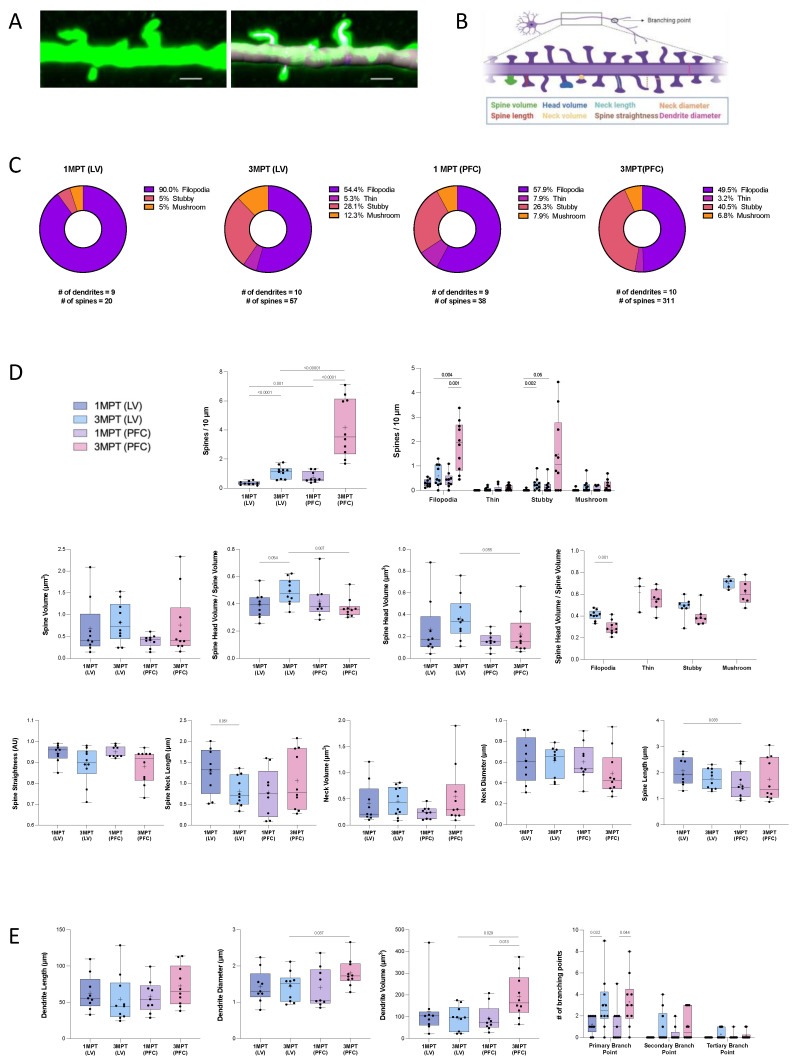
Quantitative analysis of spine and dendrite morphology in LV- and PFC-grafted NPCs at 1 MPT and 3 MPT. (**A**) Confocal image at 3 MPT of a pyramidal cell dendrite grafted in the PFC before (left) and after dendrite and spine reconstruction with the Imaris 9.5 software (right). (**B**) Schematic representation of the parameters evaluated. Created by BioRender. (**C**) Pie charts showing the distribution of the different spine categories at 1 MPT and 3 MPT in LV- and PFC-transplanted cells. Spines were classified into four different classes: filopodia, thin, stubby, and mushroom. Percentages were calculated over the total number of spines in all of the analyzed dendrites. In total 8, 8, 7, and 6 neurons, and 3, 3, 2, and 2 mice were used for 1 MPT-LV, 3 MPT-LV, 1 MPT-PFC, and 3 MPT-PFC, respectively. (**D**) Measurement of spine densities and spine morphology parameters. (**E**) Measurement of dendrite morphology parameters. Data are presented using a box and whiskers graph with plotted “min” and “max” and median values. Mean values are also indicated as “+”. Each dot represents a dendrite and spine. Spine parameters were averaged per dendrite. Statistical analyses were performed using Mann-Whitney test.

Dendritic spines are important for the establishment of most excitatory synapses and the cortical circuits. Their density and structural plasticity are thought to directly reflect the glutamatergic synaptic transmission and synaptic plasticity in the brain cortex during development, as well as the developed adult brain. Each spine may allow the neuron to control its local activity. The aim of this study was to understand the contribution of the local host environment in spinogenesis processes. Our objectives were to compare the spine maturation in human cortical pyramidal neurons derived from transplanted NPCs at 1 and 3 MPT after their in utero engraftment into the LV of the mouse embryo (E17.5); and the spine maturation after transplanting the human NPC into two distinct embryonic regions, namely the LV and the PFC, at 1 and 3 MPT. For these experiments, we used our previously reported method, which consists of the 3D reconstruction and quantitative analysis of diverse structural spine parameters on iPSC-derived pyramidal neurons [9,29] (Figure 7A; Video S6). The set of morphological parameters that have been measured in our study are depicted in Figure 7B. The diverse morphologies of dendritic spines have been reported previously with the classical distinction between filopodia, thin, stubby, and mushroom forms [54]. The spine diversity appears to be directly related to its stability and functional activity [54]. Figure 7C illustrates the proportion of the four spine categories, tested under each experimental condition. We could observe a large proportion (90%) of filopodia at 1 MPT after LV transplantation. The proportion of filopodia at 1 MPT was lower in PFC as compared to the LV transplants but remained in the major spine category (57.9%). This indicates that spines at this stage are still immature. The NPCs have moved forward to the cortical zones, and this may be the reason for the delay in the maturation process. In contrast to filopodia, the stubby and mushroom categories have been recently shown to possess a post-synaptic density with similar average protein copy numbers and topologies [55]. At 3 MPT we observed that the proportion of stubby and mushroom spines increased in the LV transplants, while only the proportion of stubby spines increased in the PFC transplants (Figure 7C). These data indicate an increase in the level of maturation until 3 MPT. We could also observe a significant increase in the total spine densities at 3 MPT compared to 1 MPT after transplantation of the NPCs into LV and PFC, with an even higher increase in the PFC-transplanted neurons compared to LV. Such increases were more pronounced for the filopodia and stubby than for the thin and mushroom categories (Figure 7D). The spine densities were very similar to those we reported previously in neonatal grafting [9], as well as those reported in a recent study using human-transplanted organoids [13]. By contrast, the total volume of spines remained unchanged over the four conditions tested. At 3 MPT, a slight but significant decrease in the relative spine head volume was observed in the PFC transplants compared to the LV transplants. This effect was predominantly observed for the filopodia spines (Figure 7D). Our results also indicate that the spine straightness and neck morphologies remained unchanged in all of the tested conditions, despite a slight decrease in the spine neck length between 1 MPT and 3 MPT in the LV transplants (Figure 7D). The total spine length was significantly lower in PFC as compared to LV at 1 MPT. We also observed a 38% increase in the total spine head volume together with a 37% decrease in the total spine neck length after transplantation within the LV. These data strongly suggest that a coordinated regulation of the neck morphology and synaptic maturation could occur and be linked to functional neuronal maturation [54]. Finally, we measured the morphological parameters of the dendrites from human cortical pyramidal neurons. While the dendrite length remained unchanged between all of the conditions tested, we could observe a significant increase in the dendrite diameter and dendrite volume, as well as the number of primary branching points at 3 MPT, when the NPCs were transplanted in the PFC compared to the LV (Figure 7E). In addition, the dendrite volume was significantly increased between 1 MPT and 3 MPT in the PFC transplants (Figure 7E). Altogether, our data show a higher complexity of dendritic arborization of the cortical pyramidal neurons when directly transplanted within the PFC compared to the LV. In parallel to higher dendritic complexity, higher spine densities were predominantly observed, while the spine intrinsic characteristics remained similar between the PFC and LV. Neuronal maturation represents a continuous process that is under the influence of local and extrinsic factors, which may affect cell migration and maturation. At the level of the LV transplants, the human-transplanted NPCs have migrated and adopted some specific trajectories toward the cortical zone before reaching a cortical destination. For the PFC-transplanted NPCs, the local and extrinsic factors were expected to increase the neuronal maturation while keeping the neotenic human phenotypes, which signifies that the xenografted cortical neurons are expected to retain some juvenile traits in adult mice [5,56].

## 4. Conclusions

Our data show that human neural precursors transplanted into the brain ventricles of embryonic immunocompetent mice can survive and differentiate into juvenile-like (not fully mature) and more mature neurons with specific patterns of migration and axonal projections over time with no abnormal proliferation, which we found reproducible and uniform among all the transplanted mice. We evaluated the functional neuronal differentiation using a detailed 3D analysis of spinogenesis. Depending on the injection site, LV vs. PFC, we discovered that the specific fetal local environments significantly modified the synaptogenesis processes while maintaining human neoteny. We are currently analyzing the molecular determinants of such processes at the level of transcriptome using single-nuclei RNA sequencing. Our in utero approach can be extended and applied in future studies using other embryonic brain regions in rodents to characterize the developmental patterns. In addition, our model enables the use of other host species for investigation and direct analyzes of the mechanisms that lead to neuropathological conditions and alterations in the development of brain circuits, with no further need for transgenic manipulations for immunosuppressive conditions at early stages of development. It also offers the possibility to investigate the possible sequelae of stem cell transplantation and provides the paths for future therapeutic approaches such as gene therapy through this technique. 

## Data Availability

Not applicable.

## Supplementary Materials

The following supporting information can be downloaded at: https://www.mdpi.com/article/10.3390/cells12071067/s1, Figure S1: Illustration of the pipeline to study neuronal projections, related to Figure 3 and Figure 4; Figure S2: Absence of Caspase-3 expression at 1 MPT and 3 MPT, related to Figure 5; Figure S3: Maturation of grafted GFP cells in LV and PFC transplants, related to Figure 5; Tables S1–S3, related to Materials and Methods Section; Video S1: Illustration of the in utero surgery with a visible embryo’s brain and injection process; related to Figure 2; Video S2: 3D representation of a GFP+ neuron in the PFC; related to Figure 3. Video S3: 3D representation of GFAP labeling, related to Figure 5; other videos available upon request; Video S4: 3D reconstruction of a dendrite segment (grey) and spines (blue) of a LV-transplanted cell for VGLUT1 labeling detection, related to Figure 5; Video S5: 3D reconstruction of a dendrite segment (grey) and spines (blue) of a PFC-transplanted cell for VGLUT1 labeling detection, related to Figure 5; Video S6: 3D reconstruction of a dendrite and the spines of a GFP+ pyramidal neuron in the PFC.
