# Supplementary material for: Developmental Changes of Human Neural Progenitor Cells Grafted into the Ventricular System and Prefrontal Cortex of Mouse Brain in Utero
Source: Cells. 2023 Mar 31;12(7):1067. doi: 10.3390/cells12071067 (PMC10093207; doi:10.3390/cells12071067)
Supplement: Supplementary file 1 [file cells-12-01067-s001.zip › MDPI_CELLS_Supplementary Materials.pdf]

Supplementary Materials

Developmental changes of human neural progenitor cells grafted into the ventricular system and prefrontal cortex of mouse brain in-utero

Figure S1

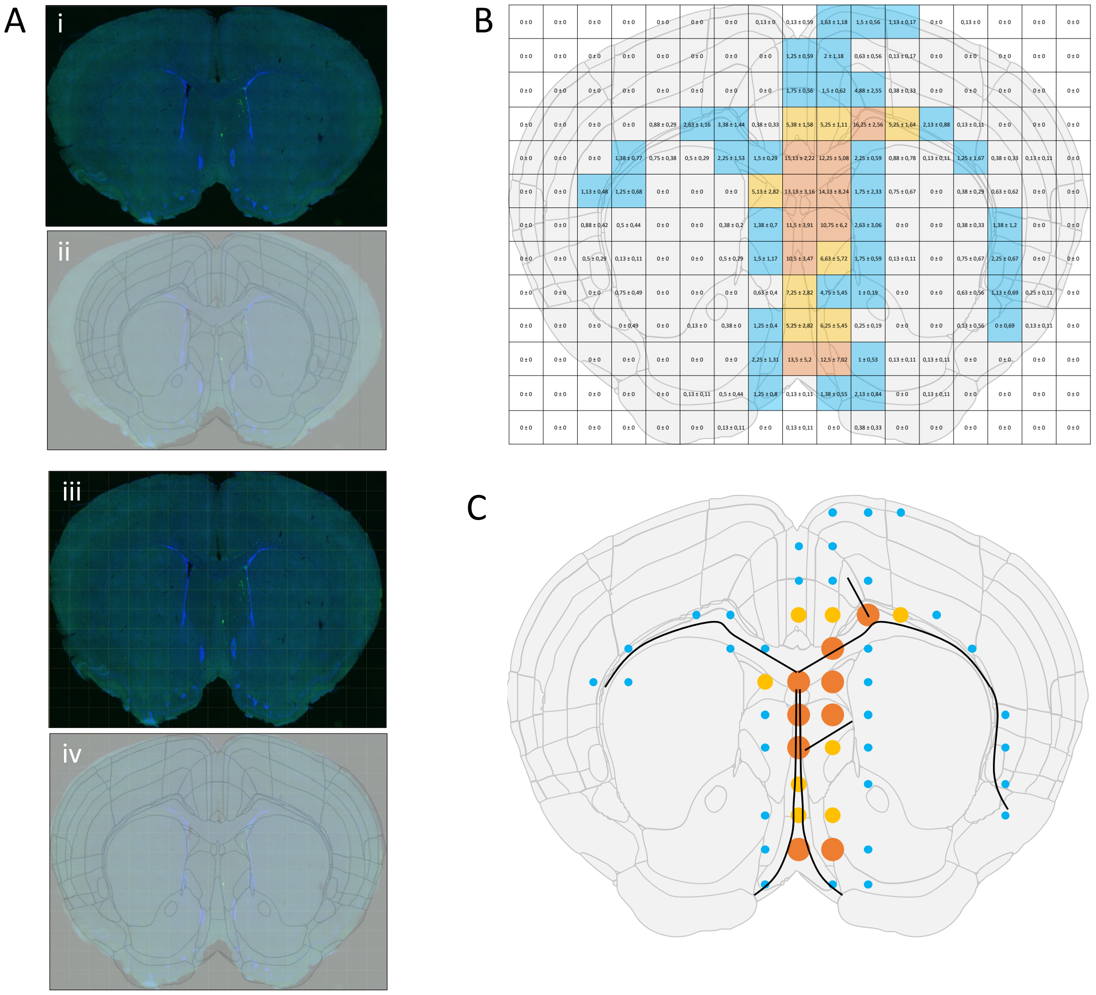

Figure S1 ***Cont.***

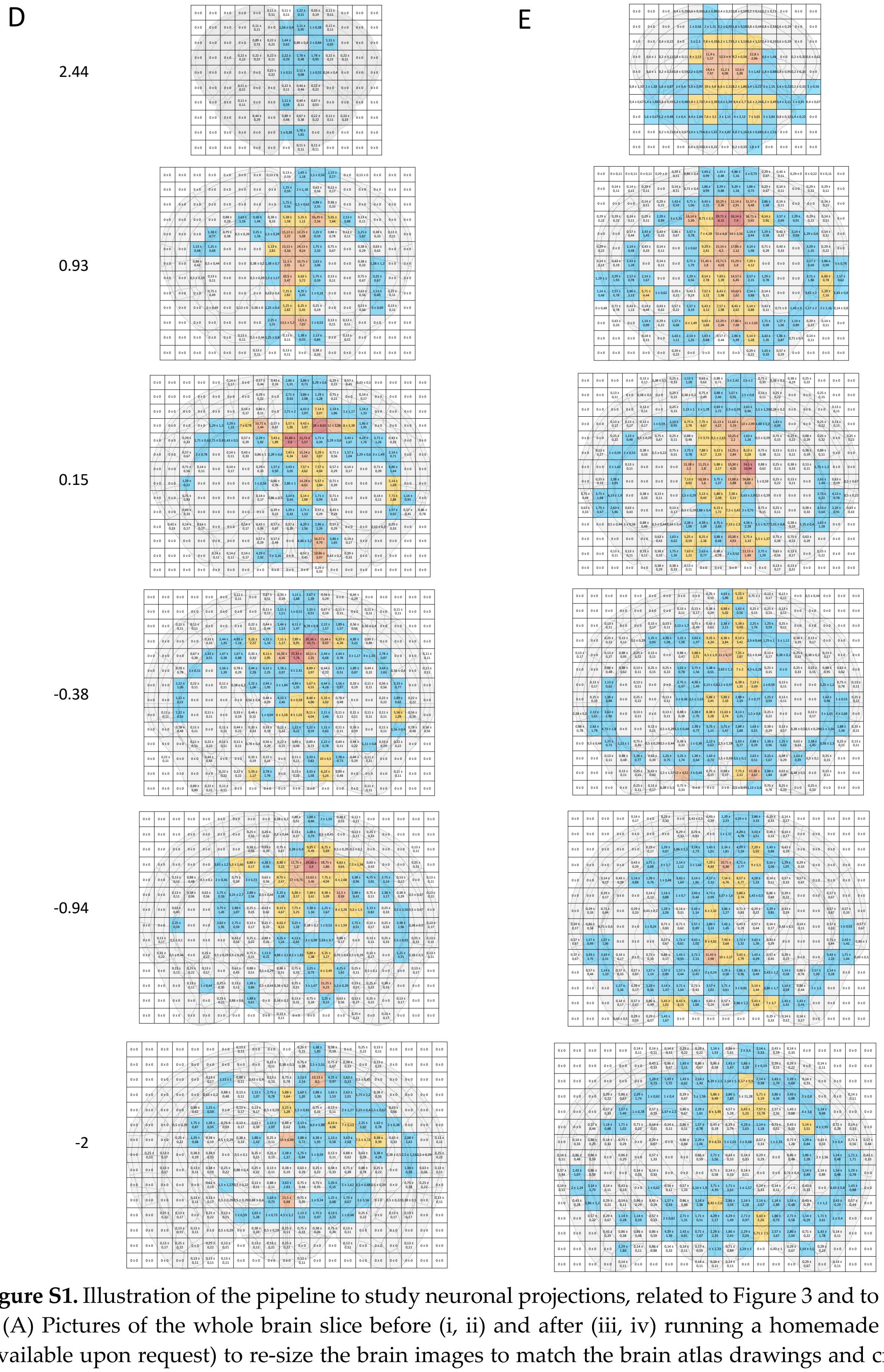

Figure S1. Illustration of the pipeline to study neuronal projections, related to Figure 3 and to Figure 4. (A) Pictures of the whole brain slice before (i, ii) and after (iii, iv) running a homemade macro (available upon request) to re-size the brain images to match the brain atlas drawings and create a grid. (B) The average and SEM of the counting of three brains are transferred into a numerical grid and merged to the coordinate illustration from the Allen Brain Atlas (Bregma, indicated in mm). A color code is used to better visualize the density and distribution of the projections. (C) Final visualization of the projection's distribution. (D-E) Extended data for the mapping of neuronal projections at 1 MPT (D) and 3 MPT (E).

Figure S2

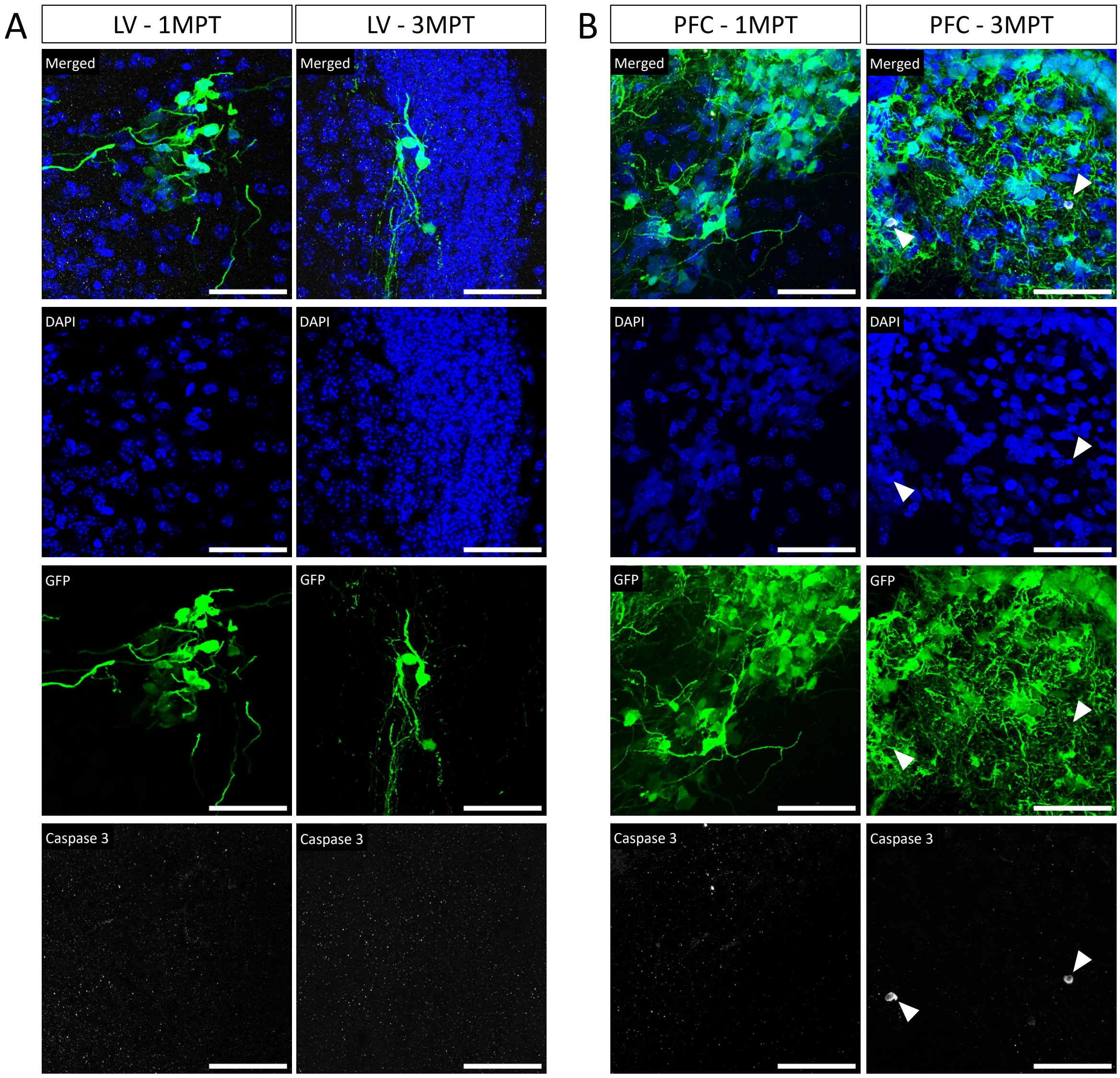

Figure S2. Absence of apoptotic GFP-cells at 1MPT and 3MPT in LV and PFC transplants, related to Figure 5. Confocal images at 1 MPT and 3 MPT for LV and PFC transplanted mice. The transplanted human cells are labeled with GFP. No GFP+ cells were labeled with the apoptotic marker Caspase3 in all conditions. Rare Caspase3+ cells (white arrows) were observed but no colocalization with the human cells is detected. Scale bar = 50µm. Results are from two independent experiments.

Figure S3

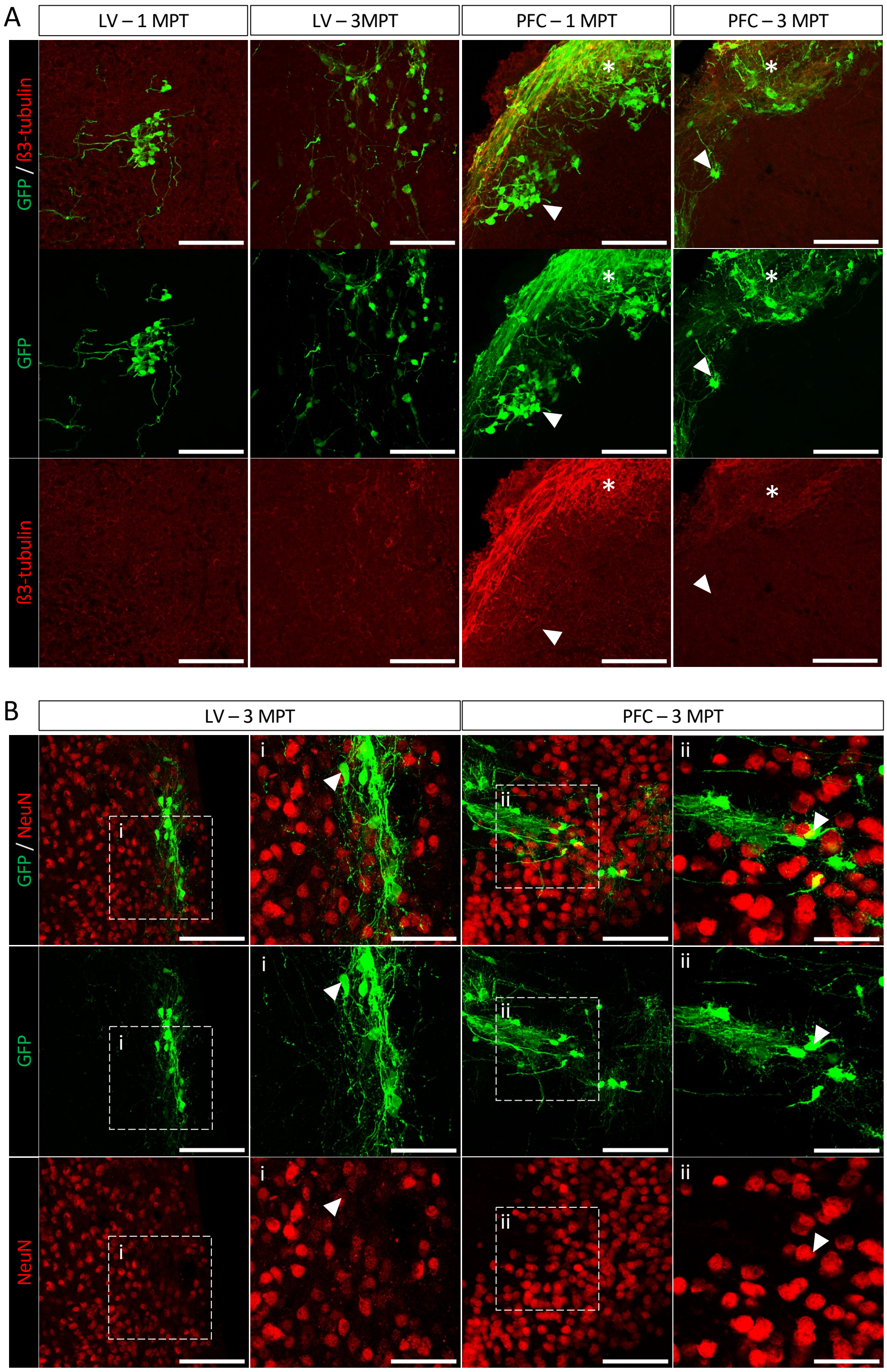

Figure S3. Maturation of grafted GFP-cells in LV (1 MPT and 3 MPT) and PFC (3 MPT) transplants, related to Figure 5. Confocal images at 3 MPT for LV and PFC transplanted mice. The transplanted human cells are labeled with GFP. (A) Immunofluorescence staining of β3-tubulin, a marker of the early phases of neuronal differentiation. (B) Immunofluorescence staining of NeuN, a post-mitotic neuronal marker. β3-tubulin-positive human cells is observed in the superficial parts of PFC transplants, where the host cells are scarce (indicated by asterisk). Only a few NeuN-positive human cells are detected within the same region. In LV transplants, only a few cells were positive for NeuN. Labeled cells are indicated by arrowheads. (A) Scale bar = 100μm. (B) Scale bar = 50μm.

Table S1. List of products with their reference and the used dilution of final concentration for NPCs medium, related to Materials and Methods Section.

NPC Medium	Ref	Company	Dilution factors
DMEM/F-12, GlutaMAXTM Supplement	31331028	ThermoFisher	1/2
Neurobasal medium	21103049	ThermoFisher	1/2
B27 supplement (50X)	11500446	ThermoFisher	1/50
N2 supplement (100X)	07152	StemCell	1/100
Penicillin Streptomycin (10 000 U/ml)	11548876	ThermoFisher	1/100
2-mercaptoethanol Stock solution (50mM)	11528926	ThermoFisher	1/1000
**Factors**	**Ref**	**Company**	**Final concentration**
Human EGF, premium grade	78006.1	StemCell	10ng/ml
Human FGF-2 IS, premium grade/BFGF	78003.1	StemCell	10ng/ml
Human Recombinant BDNF	78005	StemCell	20ng/ml
**Coating**	**Ref**	**Company**	**Final concentration**
Geltrex	A1413302	ThermoFisher	120-180µg/ml
Laminine (1mg/ml)	L2020	Sigma	10µg/ml
Poly-L-Ornithine (1mg/ml)	P3655	Sigma	15µg/ml

Table S2. List of primers used for Real-Time PCR, related to Materials and Methods Section.

Gene	Primers	Product size (bp)
Oct4	5'- CAGCAGATCAGCCACATCGC-3'	60
	3'- CCACACTCGGACCACATCCT-5'	
Nestin	5'-TTCCCTCAGCTTTCAGGACCC-3'	130
	3'-CTCAAGGGTAGCAGGCAAGG-5'	
Pax6	5'-GCAAGAATACAGGTATGGTTTTC-3'	155
	3'-GTGGGTTGTGGAATTGGTTG-5'	
Tuj1	5'-CTACAACGAGGCCTCTTCTCACAA-3'	60
	5'-GGTTCCAGGTCCACCAGAATG-3'	
VGLUT1	5'- CGACAGCCTTTTGTGGTTCC-3'	237
	3'-GGTTCATGAGTTTCGCGCTC-5'	
Cux1	5'- GCTCTCATCGGCCAATCACT-3'	75
	3'-TCTATGGCCTGCTCCACGT-5'	
CTIP2	5'-GAGTACTGCGGCAAGGTGTT-3'	98
	3'-TAGTTGCACAGCTCGCACTT-5'	
VGAT	5'-CCATCCAGGGCATGTTC-3'	103
	3'-GTGTAGCAGCACACAACG-5'	
GFAP	5'-TCCACGAGGAGGAGGTTCG-3'	200
	3'-GGCAGCAGCGTCTGTCAG-5'	
GAPDH	5'-TCTGCTCCTCCTGTTCGACA-3'	141
	3'-AAAAGCAGCCCTGGTGACC-5'	

Table S3. List of antibodies used for immunohistochemistry and immunocytochemistry, related to Materials and Methods Section.

PRIMARY ANTIBODIES				
Name	Ref	Company	Host	Dilution
GFAP	G-3893	Sigma	Mouse	1/200
GFP	A6455	Invitrogen	Rabbit	1/750
GFP	MAB3580	Millipore	Mouse	1/750
Iba1	019-19741	Wako Chemicals	Rabbit	1/500
Ki67	14-5698-80	Invitrogen	Rat	1/100
Nestin	MAB5326	Millipore	Mouse	1/200
NeuN	MAB377	Millipore	Mouse	1/200
SOX2	14-9811-80	Invitrogen	Rat	1/100
VGLUT1	135303	Synaptic System	Rabbit	1/1000
Olig2	Ab220796	Abcam	Rabbit	1/1000
ß3-Tubulin	Ab9354	Abcam	Chicken	1/200
Caspase-3	9661	Cell Signaling	Rabbit	1/300
**SECONDARY ANTIBODIES**				
Name	Ref	Company	Host	Dilution
Anti-mouse Alexa 488	715-545-150	Jackson Laboratory	Donkey	1/500
Anti-rabbit Alexa 488	711-545-152	Jackson Laboratory	Donkey	1/500
Anti-mouse Alexa 594	715-585-150	Jackson Laboratory	Donkey	1/500
Anti-rabbit Alexa 594	711-585-152	Jackson Laboratory	Donkey	1/500
Anti-rat Alexa 594	712-585-153	Jackson Laboratory	Donkey	1/500
Anti-chicken Alexa 594	703-585-155	Jackson Laboratory	Donkey	1/500
Anti-mouse Alexa 647	715-605-151	Jackson Laboratory	Donkey	1/500
Anti-rabbit Alexa 647	711-605-152	Jackson Laboratory	Donkey	1/500
Anti-rat Alexa 647	712-605-153	Jackson Laboratory	Donkey	1/500

Video S1. Illustration of the in -utero surgery with a visible embryo 's brain and injection process, related to Figure 2.

Video S2. 3D representation of a GFP+ neuron in the PFC, using Imaris 9.5 software, related to Figure 3.

Video S3: 3D representation of GFAP labeling using Imaris 9.5 software, related to Figure 5. GFAP labeling is in red fluorescence. The results show the absence of GFAP colabeling with human GFP+ cells. Other videos are available upon request.

Video S4. 3D reconstruction of a dendrite segment (grey) and spines (blue) of a LV transplanted cell, related to Figure 5. Yellow dots represent colocalization of VGLUT1 with the dendrite and the red dots represent a negative colocalization between VGLUT1 and the neuron.

Video S5. 3D reconstruction of a dendrite segment (grey) and spines (blue) of a PFC transplanted cell using the Imaris 9.5 software, related to Figure 5. Green dots represent colocalization between VGLUT1 and the spine and yellow dots represent colocalization of VGLUT1 with the dendrite. The red dots represent a negative colocalization between VGLUT1 and the neuron.

Video S6. 3D reconstruction of a dendrite and the spines of a GFP+ neuron in the PFC using the Imaris 9.5 software, related to Figure 6. Color classification of the spines is as follows: purple = filopodia, red = stubby, green = mushroom, blue = thin.
